# Bacteriophage Therapy in Freshwater and Saltwater Aquaculture Species

**DOI:** 10.3390/microorganisms13040831

**Published:** 2025-04-06

**Authors:** Deborah Albarella, Paola Dall’Ara, Luciana Rossi, Lauretta Turin

**Affiliations:** Department of Veterinary Medicine and Animal Sciences—DIVAS, Università degli Studi di Milano, 26900 Lodi, Italy; deborah.albarella@studenti.unimi.it (D.A.); paola.dallara@unimi.it (P.D.); luciana.rossi@unimi.it (L.R.)

**Keywords:** bacteria, bacteriophage, phage, therapy, alternative antimicrobials, antibacterial, multidrug-resistant, fish, shellfish, crustaceous, aquaculture

## Abstract

Bacteriophages, or phages, which are viruses with specifically restricted tropism for bacteria, have regained interest in the last few decades as alternative therapeutic agents against antibiotic-resistant pathogenic bacteria in animals and humans worldwide. In this context, bacteriophage therapy has been developed to treat bacterial infections of cultured fish, shellfish, and crustaceans. Nowadays, aquaculture is the only feasible solution to meet the continuously growing global demand for high-quality seafood. As such, it is crucial to focus on controlling the spread of pathogenic bacteria, as they have a significant economic impact on aquaculture systems. Overall, the documented research supports the application of bacteriophage therapy in aquaculture, but also underlies the need for additional studies, as it is still mostly in the scientific stage. This review aims to highlight and critically examine recent advancements in the application of bacteriophages to treat the most common bacterial infectious diseases in both freshwater and saltwater aquaculture species, providing topical perspectives and innovative advances.

## 1. Introduction

Bacteriophages (or phages) are viruses with a tropism specifically restricted to bacteria. They were used against bacterial diseases in past centuries, during the pre-antibiotic era, although their nature was unclear. Western countries abandoned their use with the advent of antibiotics, and it is only in recent years that they have been rediscovered and employed in regulated clinical trials, the results of which are well-documented in the scientific literature. This rediscovery was driven by the urgent need for alternative therapies due to the rise of clinically relevant multi-antibiotic resistance.

This paper reviews the latest advancements in developing and applying bacteriophage therapy to treat the most common and economically impactful bacterial diseases in freshwater and saltwater aquaculture species. The aims are to critically analyze the current progress and applications, show advantages and drawbacks, and highlight the gaps that need to be prioritized in future research.

## 2. Antibiotic Resistance and Bacteriophages

The widespread use and misuse of antibiotics have led to their accumulation in all habitats, contributing to the rise of antibiotic resistance as bacteria adapt to the presence of antibiotics through selective pressure [1]. Several antibiotic-resistant pathogenic bacteria have become multidrug-resistant (MDR) by acquiring antibiotic resistance genes (ARGs) to multiple antibiotics, worsening the situation. Both animals and humans play roles in creating and selecting ARGs within resistant bacterial populations. These ARGs are excreted, cross environments, and jump between species, spreading and amplifying the risks [2].

Antibiotic resistance results in therapy failure, increased morbidity, and mortality. As a consequence, healthcare costs for both humans and pets rise, along with economic losses in farming animals, posing a significant threat to global One Health. Therefore, limiting the rise of antibiotic resistance is an urgent priority. Among the various strategies to address this, such as the development of novel bioactive antibiotics, vaccines, and public awareness campaigns, bacteriophage therapy stands out as a promising solution.

Bacteriophages are viruses that specifically target bacteria and, therefore, can be used to kill bacteria without harming animal cells. As such, they represent an attractive alternative to antibiotics in treating bacterial infections in both humans and animals [3]. Bacteriophages vary greatly in size, morphology, genome, host specificity, and strain tropism (ICTV; https://ictv.global/taxonomy, accessed 21 March 2025). Depending on their replication strategy, bacteriophages are classified as lytic (virulent) or lysogenic (temperate). Lytic phages hijack bacterial cell machinery upon entry, rapidly replicate, and cause cell lysis, releasing hundreds of infectious viral particles ready to infect other host cells (productive infection). In contrast, lysogenic phages stably integrate their genome into the bacterial chromosome (or as an episome), persisting latently (as prophages) and replicating along with the bacterial chromosome. These prophages can remain dormant for extended periods before reactivating into the lytic cycle [4].

Phage therapy offers advantages over antibiotics, such as self-replication (phages multiply in the presence of bacterial targets, allowing for smaller doses and lower costs), auto-dosing (phages disappear upon target bacteria elimination), lack of interference with non-target bacteria or body cells (narrow action spectrum), and high strain specificity. However, the latter feature may be a disadvantage, which can be managed by using bacteriophage cocktails (mixtures of different phages) [5]. Additionally, phages are more environmentally friendly than antibiotics, can clear biofilms too, and, due to their short development cycles, are low-cost and easy to use and store.

However, phage therapy also has some drawbacks, such as the aforementioned narrow action spectrum, potential interference with endogenous phages, and the possible emergence of newly acquired bacterial resistance to phages, which could reduce therapeutic effectiveness. Despite this, phages co-evolve with their hosts, and counter-adaptation to developed resistance is likely to occur, ultimately benefiting bacteriophage therapy in the long term.

To improve bacteriophage therapy, it is essential to expand the understanding of phages, particularly through the annotation of complete DNA genome sequences for accurate identification, prediction of transduction efficiency, and avoidance of undesirable elements. Additionally, it is also necessary to clarify the mechanisms behind the emergence of phage resistance, which are still largely unknown.

## 3. Phage Therapy in Aquaculture

Concurrent with the increasing number of publications on the potential therapeutic applications of bacteriophages to treat human bacterial infections, the use of phages in veterinary medicine has also increased, especially for livestock, poultry, and aquaculture species. While antibiotics have proven effective in managing bacterial infections in aquatic species, their widespread use has contributed significantly to the global health problem of antimicrobial resistance. As a result, reducing or even avoiding antibiotics in aquaculture is now strongly recommended.

In 2020, the aquaculture sector accounted for 49.2% of global fish production. According to the latest FAO Report, in 2022, global fisheries and aquaculture production increased by 44% compared to 2020, reaching a total output of 223.2 million tons of aquatic animals (FAO; https://www.fao.org/newsroom/detail/fao-report-global-fisheries-and-aquaculture-production-reaches-a-new-record-high/en accessed 21 March 2025). However, bacterial diseases remain the major challenge in aquaculture, resulting in huge economic losses. Aquatic animals are susceptible to numerous bacterial infections or co-infections, as they are exposed to a wide variety of pathogenic microorganisms in their environment. Table 1 and Table 2 summarize the most frequent fish bacterial infections and co-infections, along with the corresponding diseases [6,7].

In the absence of effective vaccines that could prevent the majority of diseases affecting aquatic species, or due to the practical impossibility of vaccinating fish at early life stages because of their small size or immature immune system [8], phage therapy plays the dual role of therapeutic and preventive measures against bacterial infections in aquaculture. This is especially important during larvae production, before the fish are introduced into aquaculture containers [9]. Furthermore, phages can effectively lyse bacteria in both planktonic and biofilm states.

Phage therapy can be approached in two ways: “sur mesure” (tailored for individual cases) and “prêt-à-porter” (a universal, one-size-fits-all method). The “sur mesure” approach involves selecting and applying specific phages to target pathogens that have been previously isolated from the patient or environment. In contrast, the “prêt-à-porter” approach involves developing a phage cocktail that is prepared for immediate use and contains multiple phages capable of lysing pathogens commonly encountered in specific types of infections [10]. Regardless of the approach, the isolation, identification, whole genome sequencing, and genomic analysis of bacteriophages are essential for obtaining detailed information about the phages and establishing phage libraries. These libraries are necessary for preparing effective phage cocktails specifically tailored to pathogens identified by local farmers in a target region. Genetic engineering techniques, synthetic biology, and in vitro genome assembly are valuable tools for modifying phages and overcoming phage resistance. Additional challenges include ensuring the efficient delivery of phages at high concentrations to the infection site and addressing potential immune-mediated phage clearance.

Phage therapy is crucial for reducing mortality due to bacterial infections in aquaculture. Successful application requires careful planning of the timing, dose, and frequency of phage delivery, taking into account the bacterial pathogen’s virulence characteristics, the nature of the outbreak, and the temporal dynamics of the major pathogenic bacteria. In this context, phage therapy is most effective during spring when aquatic pathogenic bacteria exhibit the highest diversity, which correlates with a higher risk of outbreaks [11]. Several factors influence the efficacy of phage therapy in aquaculture, including the fish’s weight, the required phage dose, early disease diagnosis, and environmental conditions. These conditions encompass the physical and chemical characteristics of the water, such as salinity, organic matter content, temperature, and pH. In particular, the efficacy of phage therapy increases in the presence of high salt content, making it particularly suitable for marine aquaculture systems and potentially for non-marine aquatic systems alike [12]. Preliminary in vitro examination of phage–bacteria interactions is crucial for ensuring specificity for the target pathogen, avoiding any negative impact on the entire bacterial community, determining the multiplicity of infection (MOI), and therefore, establishing the appropriate phage dose. This is vital for optimizing the protocol to be tested in aquaculture systems. Lastly, maintaining the viability of bacteriophages in water, particularly ensuring high concentrations over extended periods, is an essential aspect to consider [13].

The selection of the phage delivery route depends on the nature of the infection, the size of the farm, the cost of phage preparation, and the fish species. The three primary methods for administering phages in aquaculture are immersion, injection, and oral administration. Immersion is less time-consuming, while injection, which is ideal for systemic infections, is more time-consuming and invasive, potentially causing high mortality. Oral administration is considered the most practical method due to its low cost and minimal stress on the fish. However, challenges include the loss of phage stability and the acidic, proteolytic conditions in the gut, which may require coating systems or the addition of acid neutralizers to the phage suspension. Bacteriophages should be able to endure dry conditions on feed following coating for oral feed applications. Longer phage survival is critical for reducing feed preparation costs and maximizing commercial benefits. Recirculating aquaculture systems (RAS) have recently gained attention because they recycle water after treatment, reducing the amount of water needed for fish farming. This method is advantageous for both phage delivery, as their small size enables them to remain in the system for protracted periods, thereby providing prolonged protection, and guaranteeing the biosafety of such a system, which is considered a vulnerable environment for bacterial disease outbreaks [14].

### 3.1. Phage Therapy in Fish Culture

**Figure 1:**
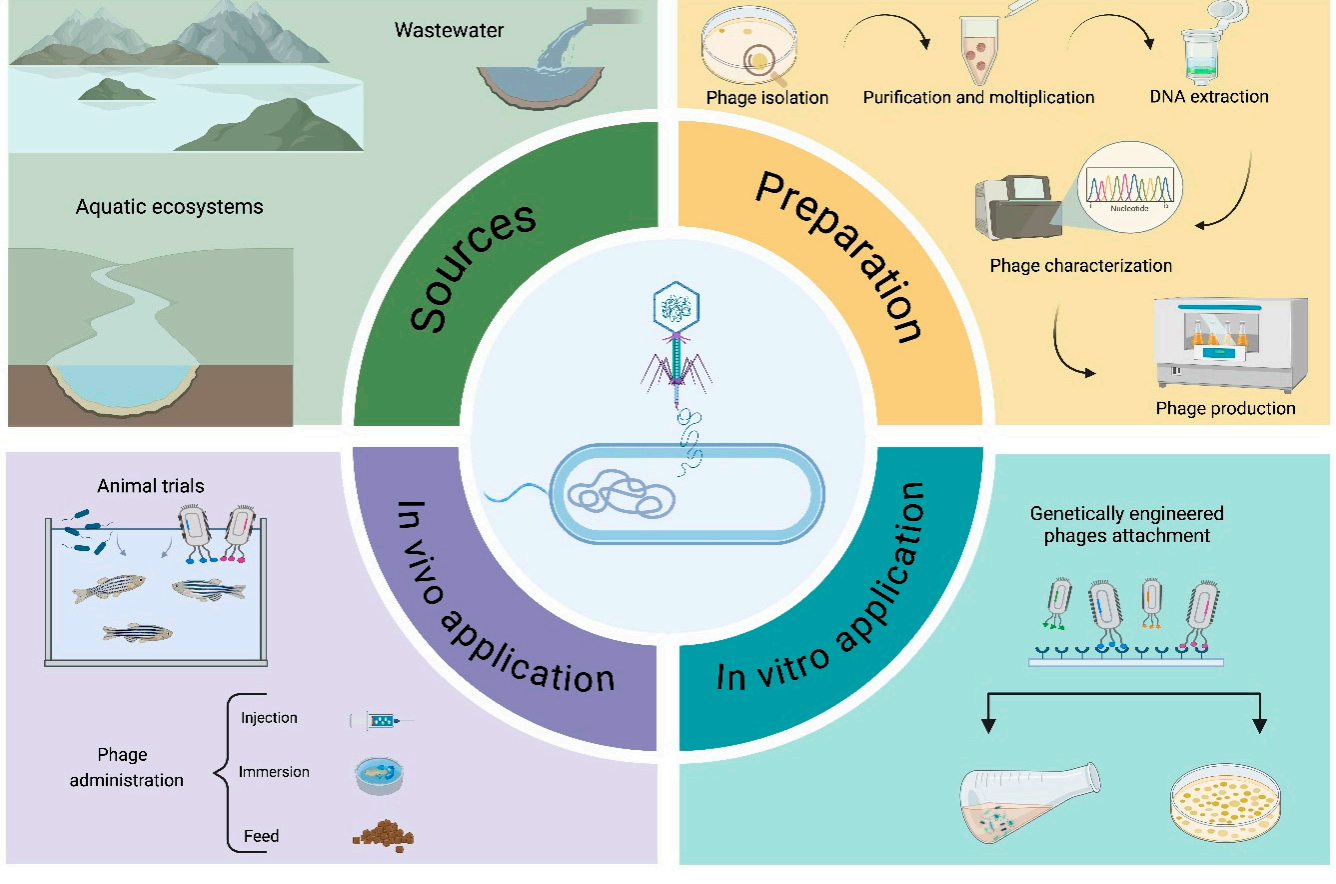
Bacteriophage therapy in aquaculture. Phages are collected from natural and wastewater sources, isolated and characterized in the laboratory, and engineered before being tested in vitro. The selected modified phages are then tested in vivo on infected animals.

Aquaculture wastewater and the environment have both been found to contain numerous antibiotic-resistant genes and antibiotic-resistant bacteria [15]. As a result, bacteriophages may be employed as alternatives to antibiotics, helping to reduce antibiotic usage at the source and address concerns related to antibiotics (Figure 1). However, currently, phages are primarily used in research at the bench level or in small-scale trials to prevent and control antibiotic-resistant bacterial infections in aquaculture [16]. As reported below, research on the interactions between phages and their aquatic hosts has mainly been conducted in marine environments, with relatively little focus on freshwater ecosystems. Zebrafish are commonly used as model species for toxicological assessments and environmental monitoring due to their rapid result evaluation and the ease of breeding. Other fish species have also been experimentally treated to address infections caused by the most common bacterial pathogens in aquaculture. In particular, research has focused on some bacterial species belonging to the genera *Aeromonas*, *Vibrio*, *Edwardsiella*, *Streptococcus*, and *Flavovacterium*, which are particularly pathogenic for fish, mollusks, and crustaceans. Less information is available on *Citrobacter*, *Pseudomonas*, *Yersinia*, *Plesiomonas*, and *Photobacterium*, although these pathogens can still cause significant economic damage to the aquaculture industry. *Aeromonas* spp. is the pathogen for which the highest number of in vivo trials have been performed, followed by *Vibrio* spp., particularly in larvae models.

#### 3.1.1. *Aeromonas*

*Aeromonas* spp. includes various Gram-negative bacterial species that are pathogenic for freshwater fish and other animal species. It is a zoonotic pathogen and may also be involved in biofilm formation. While some studies have focused on using a single phage against *Aeromonas* spp., others have demonstrated that using a cocktail of phages is more effective for resolving infections. The most common infectious diseases in aquaculture are caused by *A. hydrophila* and *A. salmonicida.*

*A. hydrophila* is widely distributed in aquatic environments, where it causes hemorrhagic septicemia, red sore disease, and ulcerative syndrome in fish, particularly in carp and catfish. Antibiotic-resistant strains lead to high mortality rates and significant economic losses. As a result, several phages with potential for phage therapy (e.g., lytic and bacteria-killing properties, suitable host spectrum, stability across a wide range of pH and temperatures, short latency, and lack of integrating elements) against *A. hydrophila* have been isolated and characterized in vitro [17,18,19].

Several studies have confirmed the efficacy of single phages in treating *A. hydrophila* infection in fish, such as the cyprinid loach, striped catfish, tilapia, carp, and the zebrafish model. Table 3 summarizes, for the in vivo studies, the dosages of bacteria and phages administered via different routes in various fish. A single administration (injection or oral) of either phage pAh1-C or pAh6-C (classified morphologically as Myoviridae) to cyprinid loaches increased survival rates against *A. hydrophila* challenge, despite the overall considerable mortality rate [20]. Similarly, promising results were obtained in striped catfish following oral administration of the phage PVN02 in surface-sprayed pellets [21], and in Nile tilapias after immersion exposure to the wide-spectrum lytic bacteriophage pAh6.2TG [22]. In tilapia, the Myoviridae bacteriophage PAh4 also reduced mortality due to *A. hydrophila* when administered with the diet [23]. Phage Akh-2, belonging to the Siphoviridae family, administered by immersion in loaches, demonstrated a more extended survival (over 40%) following the bacterial challenge [24]. Trials on carp intraperitoneally injected with bacteriophage PZL-Ah152 not only confirmed the efficacy of single-phage therapy in protecting against MDR *A. hydrophila* but also showed that the fish gut microbiota equilibrium was not disrupted [25]. The single bacteriophage Ahy-Yong1 exhibited specific activity against one of 35 tested multidrug-resistant *A. hydrophila* strains, and after intraperitoneal injection in experimentally infected carp, it reduced mortality to 20–40% depending on the administration time (better for prophylaxis) [26]. This phage also proved effective in eliminating the biofilm produced by the same specific MDR strain of *A. hydrophila* [26]. In the zebrafish model, two different phages classified as Myoviridae showed similar potential for controlling *A. hydrophila*. The intraperitoneally injected lytic phage pAh-1 improved the survival rate by 43% after infection with *A. hydrophila* [27], while the bacteriophage AhMtk13a, administered by immersion, showed variable efficacy depending on the administration route of the challenge bacteria (immersion, immersion following dermal abrasion, or intraperitoneal injection) [28]. A recently identified phage, phiA034 (assigned to the Casjensviridae family), showed promising properties in vitro, such as targeting several (14) species of *Aeromonas*, including *A. hydrophila*, having a short latency (20 min), and exhibiting high tolerance to temperature (30 to 70 °C) and pH (6 to 10) variations [29]. However, in vivo trials are still needed.

Two other significant fish pathogens of the *Aeromonas* genus are *Aeromonas salmonicida* and *Aeromonas schubertii*. *Aeromonas salmonicida* can infect a variety of commercial fish species, including turbot, salmon, Atlantic cod, rockfish, sea bream, and sole, causing furunculosis. This disease leads to inflammation in the liver, kidney, and spleen, as well as muscle granuloma and skin ulceration. Three newly isolated virulent phages (ZHA, ZHD, and ZHF) were tested for their antibacterial activity in turbot, and one of them (ZHF) demonstrated robust activity both in vitro and in vivo following intraperitoneal injection [30]. Another study demonstrated the safety and effectiveness of Phage AS-A in controlling *A. salmonicida* when administered by immersion to juvenile Senegalese sole [31]. The lesser-known *Aeromonas schubertii* is a significant opportunistic pathogen that can infect an increasing range of aquatic animals, including snakehead, Nile tilapia, Garra rufa, rainbow trout, mandarin fish, prawns, and zebrafish. Recently, a bacteriophage, SD04, a lytic phage from the Siphoviridae family, was isolated, characterized, and tested against *A. schubertii* infection. This bacteriophage significantly increased survival rates when administered by intraperitoneal injection (83%) or by immersion (100%) in snakehead fish challenged with pathogenic *A. schubertii*, and it also inhibited bacterial multiplication in the liver [32].

**Phage cocktails**, composed of two or more bacteriophages, have been evaluated to overcome the limitations of using a single phage to target *Aeromonas hydrophila.* Typically, the efficacy of phage therapy is enhanced by using a cocktail of bacteriophages due to their synergic action, extended host range, and reduced likelihood of phage resistance developing in bacteria. However, one study (limited to in vitro only) reported that utilizing a cocktail of phages (TG25P and CT45P) targeting different receptors on the same bacterium does not necessarily result in greater efficacy than using a single phage [33].

A combination of two mixed phages (vB_ AhaP_PZL-Ah8 and vB_ AhaP_PZL-Ah1) demonstrated more effectiveness than single phages both in vitro and in vivo in substantially reducing bacteremia in carp intraperitoneally injected with *A. hydrophila*, as well as in inhibiting biofilm formation [34]. Another two-phage cocktail (D6 and CF7) was tested in phage-coated feed to protect carp against *A. hydrophila*. Both phages were able to survive in the fish intestine and reach the kidney following oral administration, resulting in a substantial reduction in fish mortality in a dose-dependent manner [35]. Even better results (up to 100% survival rate) were achieved in striped catfish by injecting a phage cocktail consisting of Φ2 and Φ5 [36]. Finally, BAFADOR^®^, a bacteriophage cocktail consisting of seven bacteriophages (three targeting *A. hydrophila* and four targeting *Pseudomonas fluorescens*), was tested in rainbow trout via immersion. It proved effective for both therapeutic and prophylactic applications and also activated the nonspecific immune system responsible for clearing various bacterial infections [37].

A phage cocktail consisting of two to five lytic bacteriophages (HER98, HER110, SW69-9, L9-6, and Riv-10) belonging to the Myoviridae family proved effective against *A. salmonicida* in vitro, except against strains carrying Prophage 3 [38]. This highlights the importance of studying bacteriophage–bacteria interaction as a first step in selecting viral candidates potentially effective for phage therapy. More recently, the same research team reported the discovery and characterization of a novel virulent bacteriophage (MQM1, belonging to the Podoviridae family), which is specific for *A. salmonicida* strains carrying Prophage 3, making it resistant to the previous phage cocktail [39]. Including this new phage in a cocktail may overcome resistance issues and hold promise for preventing furunculosis.

Bacteriophages are also effective in preventing and clearing *Aeromonas hydrophila* **biofilms**. In this context, phages G65, W3, and N21 (belonging to the Myoviridae family) and Y71 and Y81 (belonging to the Podoviridae family) demonstrated a reduction in mature biofilms of more than 60% by using in vitro biofilm formation and clearance assays. Specifically, phages G65 and Y81 showed considerable inhibition of biofilm formation, G65 and W3 exhibited strong biofilm-clearance effects, while N21 and Y71 displayed similar bacteriolytic activities and biofilm-inhibiting effects. In the presence of bacteriophage G65, almost no biofilm could be detected [17].

A phage cocktail consisting of four bacteriophages (vB_AehM_DM8, vB_AehM_DM12, and vB_AehM_DM14 from the Myoviridae family, and vB_AehP_DM11 from the Podoviridae family) was used to compare the efficiency of different **delivery routes** (intraperitoneal injection, immersion, and oral) in Nile tilapia [40]. All the routes were effective against *Aeromonas* infection; however, intraperitoneal injection proved to be the most effective for both systemic and localized infections [40]. The bath route was the least effective in treating hemorrhagic septicemia due to the rapid progression of the disease, with efficacy limited to infections concentrated on the epidermis, scales, and gills of fish [40]. The oral route is preferred for infections confined to the gastrointestinal tract; however, it also proved effective in reducing *A. hydrophila* mortalities [35,40].

**Table 3:** Summary of dosages of bacteria and phages administered via different routes to various fish against *Aeromonas* spp.

Bacterium (*Aeromonas*) Strain/ Administration Route/Dosage (CFU)	Phage Acronym/Administration Route/Dosage (PFU)	Fish	Reference
*A. hydrophila* isolated from diseased fish or environmental/intraperitoneal injection/2.6–26 × 10^6^/fish	pAh-1-C or pAh6-C/intraperitoneal injection–oral/1.7–3 × 10^7^/fish	Cyprinid loach	[20]
*A. hydrophila* 4.4T isolated from diseased fish/immersion/10^3^–10^7^/mL of water	PVN02/oral/10^4^–10^6^/g of pellet	Striped catfish	[21]
*A. hydrophila* BT14 isolated from diseased fish/immersion/2 × 10^7^/mL of water	pAh6.2TG/immersion/2–20 × 10^6^/mL of water	Nile tilapia	[22]
*A. hydrophila* UR1 isolated from diseased fish/intraperitoneal injection/3.16 × 10^5^/fish	PAh4/oral/10^5^–10^8^/g of diet	Tilapia	[23]
*A. hydrophila* KCTC2358 from Korean Collection/immersion/10^6^–10^8^/mL of water	Akh-2/immersion/10^8^/mL of water	Loach	[24]
*A. hydrophila* 152 multidrug-resistant/intraperitoneal injection/2 × 10^8^/fish	PZL-Ah152/intraperitoneal injection/2 × 10^9^/fish	Crucian carp	[25]
*A. hydrophila* A18 isolated from market aquatic produce/intraperitoneal injection/10^7^/fish	Ahy-Yong1/intraperitoneal injection/10^6^/fish	Brocade carp	[26]
*A. hydrophila* KCTC12487 from Korean Collection/intraperitoneal injection/2 × 10^6^/fish	pAh-1/intraperitoneal injection/2 × 10^7^/fish	Zebrafish	[27]
*A. hydrophila* GW3-10 isolated from diseased trout/immersion–immersion following dermal abrasion–intraperitoneal injection/10^5^–10^7^/mL of water—10^7^/mL of water after dermal abrasion—10^2^–10^7^/fish	AhMtk13a/immersion/10^8^/mL of water	Zebrafish	[28]
*A. salmonicida* AS01 isolated from diseased turbot/intraperitoneal injection/8 × 10^4^/fish	vB_AsM_ZHF/intraperitoneal injection/8 × 10^2^–10^6^/fish	Turbot	[30]
*A. salmonicida* CECT894 from Collection/immersion/10^8^/mL of water	AS-A/immersion/10^10^/mL of water	Sole Senegalese juvenile	[31]
*A. schubertii* GC1 islated from diseased snakehead/intraperitoneal injection–immersion/1.5 × 10^2^/fish-10^5^/mL of water	SD04/intraperitoneal injection–immersion/1.5 × 10^4^/fish-10^7^/mL of water	Snakehead	[32]
*A. hydrophila* Ah-138//intraperitoneal injection/2 × 10^6^/fish	vB_ AhaP_PZL-Ah8 and vB_ AhaP_PZL-Ah1(cocktail)/intraperitoneal/10^4^/fish	Crucian carp	[34]
*A. hydrophila* MTCC1739 from Collection/intraperitoneal injection/10^6^–10^7^/fish	D6 and CF7 (cocktail)/oral/10^6^–10^8^/g of feed	Carp	[35]
*A. hydrophila* N17/intraperitoneal injection/3.2 × 10^6^/fish	Φ2 and Φ5 (cocktail)/injection/10^5^–10^7^/fish	Striped catfish	[36]
*A. hydrophila* and *P. fluorescens*/intraperitoneal injection/0.2 mL of the [1.5] mL/fish	BAFADOR^®^ cocktail (7 phages)/immersion/10^5^/mL of water	Rainbow trout	[37]
*A. hydrophila* 10098 from Philippine National Collection/intramuscular injection/2 × 10^6^/fish	vB_AehM_DM8, vB_AehM_DM12, vB_AehM_DM14 and vB_AehP_DM11 (cocktail)/injection–immersion–oral/4 × 10^9^/fish-6.1 × 10^6^/mL of water-1.2 × 10^10^/g of pellet	Nile tilapia	[40]

#### 3.1.2. *Vibrio*

*Vibrio* spp. are Gram-negative, curved, motile bacilli present in marine, estuarine, and freshwater environments. They are responsible for vibriosis, the most common disease of marine and estuarine fish both in natural and farm systems worldwide. However, *Vibrio* spp. infections can also affect freshwater fish. The genus Vibrio includes pathogens that cause a range of devastating diseases, often resulting in up to 100% mortality in contaminated facilities. This leads to significant impacts on aquatic ecosystems and causes substantial economic losses globally [11]. Moreover, *Vibrio* spp. are capable of forming biofilms and exhibit resistance to several antibiotics. As a result, recent research has focused on expanding the phage library against *Vibrio* spp., enriching it with newly isolated lytic and lysogenic bacteriophages that could be potentially useful in aquaculture. Lytic bacteriophages IKEM_vK, IKEM_v14, and IKEM_v5, isolated from sea bream aquaculture water, demonstrated a broad host range against various *Vibrio* species, stability at pH variations (6 to 11), and temperatures up to 60 °C [41]. These phages were shown to lyse planktonic *Vibrio* spp. and inhibit biofilm formation, making them promising candidates for use in aquaculture [41]. These in vitro characteristics support their potential for in vivo application.

Table 4 summarizes, for the in vivo studies, the dosages of bacteria and phages administered via different routes in various fish against *Vibrio* spp.

*V. harveyi* is the most commonly isolated *Vibrio* species from cultured fish, with clinical manifestations varying depending on the aquatic species [11]. These manifestations include ocular lesions, blindness, gastroenteritis, muscular necrosis, dermal ulcers, and tail rot in fish, as well as luminous vibriosis and acute hepatopancreatic necrosis disease in shrimp, often leading to mass mortality [42]. This pathogen significantly impacts the economics of fish aquaculture, particularly in groupers, gilthead seabream, sole, and European seabass [43]. The recently identified phage vB_VhaS_R21Y (R21Y) (Siphoviridae) showed relative stability under varying pH and temperature conditions and demonstrated high species-specificity in vitro for *V. harveyi* [42]. However, its low lysis efficiency, the need for high MOI, and the development of bacterial resistance over time indicate that further research is required before it may be considered for in vivo use. The lytic phage VhKM4 (Myoviridae) demonstrated virulence against both pathogenic *V. harveyi* and *V. parahaemolyticus*. However, additional studies are necessary to explore its genomic characteristics, environmental resilience, and the phage life cycle before assessing its potential for in vivo application [44]. A jumbo lytic bacteriophage, vB_VhaM_pir03 (also Myoviridae), was identified as a promising candidate for phage therapy against *Vibrio harveyi* due to its rapid adsorption, short latent phase, broad activity against *V. harveyi* strains, and other antibiotic-resistant *Vibrio* species. It also lacks transduction potential, virulence genes, or antibiotic-resistance genes, making it an attractive option for further research in phage therapy [45]. In vivo trials have been conducted not in fish, but in shellfish (see later). The same research group subsequently identified another phage, Vibrio phage Virtus (Siphoviridae), which exhibited similar features, including infectivity across multiple *Vibrio* species, lack of transduction potential, virulence or antibiotic-resistance genes, and a rapid replication cycle [46]. This bacteriophage was tested in vivo in gilthead seabream larvae challenged with pathogenic *V. harveyi*, and it proved effective in significantly increasing the survival of the larvae [46]. Furthermore, the same research group recently identified another bacteriophage, vB_VhaS_MAG7 (Siphoviridae), which also targets *V. harveyi* but with greater strain specificity. Like the other phages, it lacks transduction potential, virulence or antibiotic-resistance genes, features a quick replication cycle, and demonstrates a strong lytic activity [43]. This phage was tested in vivo in gilthead seabream larvae challenged with the specific pathogenic *V. harveyi* strain and significantly improved larval survival, with no observed toxicity, demonstrating a safe profile [43].

Other studies have focused on bacteriophages targeting *V. anguillarum* and *V. parahaemolyticus,* both of which can infect fish.

*V. anguillarum* is a marine pathogenic species responsible for hemorrhagic septicemia vibriosis in fish, mollusks, and crustaceans. Among various phages isolated to target pathogenic strains of *V. anguillarum*, the CHOED bacteriophage (Podoviridae) was selected, characterized, and tested in vivo through immersion in salmon. This phage demonstrated up to 100% survival in experimental infection with pathogenic *V. anguillarum* [47]. The same research group later sequenced the entire phage genome [48]. Another team focused on the wide host-range phage KVP40 and helped clarify the resistance strategies developed by different *V. anguillarum* strains against bacteriophages. The study identified multiple mutational and non-mutational resistance strategies, including nucleotide stop or frameshift mutations, receptor amino acid changes or downregulation, abortive infections, and biofilm formation [49]. The complexity and variety of mechanisms identified highlight the importance of gaining deeper insights and carefully selecting effective phages before conducting in vivo trials.

*V. parahaemolyticus*, recognized for a long time as a pathogen for shrimps, has more recently been identified as a pathogen for finfish as well. It is a major cause of seafood-borne disease. A lytic bacteriophage specific to *V. parahaemolyticus*, VpKK5 (Siphoviridae), was isolated and characterized in vitro for potential therapeutic application in aquaculture [50]. This phage demonstrated complete lytic activity with a narrow host range, restricted to *V. parahaemolyticus* only. It also showed no horizontal gene transfer mechanisms, lysogeny, or virulence genes. Additionally, the phage has a short latent period, is tolerant to temperatures up to 40 °C, and can withstand a wide range of pH [50]. Given these features, VpKK5 could be a promising candidate for phage therapy in aquaculture, especially in natural (tropical) environments where the water temperatures fluctuate around 30 °C.

Considering the high degree of genotypic and phenotypic diversity among pathogenic *Vibrio* spp., the potential use of a phage with a broad host range, effective against multiple species simultaneously, can be highly beneficial in aquaculture. In this context, during a screening of numerous phages, the lytic bacteriophage vB_VhaS-R18L (R18L) (Siphoviridae) was identified as having a wide host range [51]. It proved effective in vitro against several strains from at least five different *Vibrio* species (*V. harveyi*, *V. parahaemolyticus*, *V. alginolyticus*, *V. cholera*, and *V. proteolyticus*). The phage was characterized by a short latency, absence of lysogeny and virulence genes, and stability across pH variations from 6 to 11 and temperatures ranging from 4 to 50 °C [51]. Given these features, vB_VhaS-R18L could be a promising and safe candidate for bacteriophage therapy.

A specific approach was developed using two lytic bacteriophages (φSt2 and φGrn1), both specific to *V. alginolyticus*, which were administered together to live preys such as the crustaceous Artemia salina to protect fish against *Vibrio alginolyticus* infections (see later) [52].

As bacteria develop resistance to phages, **phage cocktails** offer a more effective strategy to counteract this issue. However, the random selection of phages for cocktails can be labor-intensive and time-consuming. A study proposed a strategy for the rapid selection of phages and the preparation of cocktails based on the use of bacteriophage-resistant bacteria, which evolved quickly by exposure to a single bacteriophage as hosts for identifying other co-present bacteriophages with different infection strategies [53]. Using this method, the researchers prepared a phage cocktail containing bacteriophages with different infection strategies to avoid cross-resistance. They isolated two lytic phages (Va1 and Va2) that infect *V. alginolyticus* and tested them both alone and in combination. When the target bacterium developed resistance, they isolated a third phage (Va3), a jumbo phage, which was more effective in preventing the emergence of phage-resistant bacteria compared to the cocktail containing only the first two phages. Finally, they isolated VP4, and when added to the cocktail, the combination containing VP4 proved even more effective [53].

**Fish larvae** have sometimes been used just as models for testing bacteriophages in vivo, as reported above [43,46]. However, mortality due to *Vibro* spp. can also occur during early life stages due to detrimental interactions between fish larvae and bacterial communities in the surrounding water. Larvae may be infected by the eggs, water, or initial feed. Phage treatment can be administered directly to the water in larval cultures offering the double advantage of avoiding phage inactivation by the host immune system (not fully mature at the larval stage) and controlling the pathogen not only within the larvae but also in the surrounding water where the bacteriophage is released. This helps limit potential bacterial colonization. A study in the zebrafish model demonstrated the efficacy of phage VP-2 (administered via immersion) in protecting larvae from *V. anguillarum* infections, significantly improving the survival rate of infected fish larvae [9]. Another study, conducted in the absence of bacterial challenge, showed that administering the broad-host-range phage vB_Pd_PDCC-1 by immersion to longfin yellowtail larvae from the egg stage up to 12 days post-hatching increased larval quality, survival, and growth [54]. At this stage, bacteriophage therapy serves not only as a potential biological control measure but also as a means to enhance ontogenetic development.

In turbot and cod larvae, the broad-host-range phage KVP40, delivered by immersion, was able to infect at least eight species of *Vibrio* spp. (*V. anguillarum*, *V. parahaemolyticus*, *V. alginolyticus*, *V. cholera*, *V. natriegens*, *V. mimicus*, *V. splendidus*, and *V. fluvialis*), as well as *Photobacterium leignathi*. The phage significantly delayed or reduced mortality following the challenge with four distinct strains of *V. anguillarum*, and mitigated larval mortality caused by the background population of other pathogens [55].

Using bacteriophages directly during the larval stage can be highly beneficial. Indeed, reducing, but not completely eradicating the vibrios may be advantageous. This approach allows the maturing fish’s immune system to be exposed to low levels of bacteria, helping to develop immunity over time. In conclusion, phage therapy has shown promising results in treating fish larvae within aquaculture systems, offering a potential alternative to conventional methods.

Since **biofilm formation** is correlated with increased bacterial pathogenicity and disease recurrence, there is an urgent need to disperse biofilms. The bacteriophage pVa-21 (classified as Myoviridae) showed lytic activity against both planktonic and biofilm *V. alginolyticus*, and inhibiting activity of bacterial growth in both states [56]. The lytic phage vB_VpaP_FE11 (FE11) (Podoviridae) demonstrated efficient prevention of *V. parahaemolyticus* biofilm formation in vitro, along with features that are favorable for bacteriophage therapy, including stability at temperatures ranging from 20 to 50 °C and pH from 5 to 10, as well as a short latent period [57]. Additionally, the above-mentioned lytic bacteriophages IKEM_vK, IKEM_v14, and IKEM_v5 proved capable of lysing planktonic *Vibrio* spp. and inhibiting biofilm formation by multiple *Vibrio* species [41].

**Table 4:** Summary of dosages of bacteria and phages administered via different routes to various fish against *Vibrio* spp.

Bacterium (*Vibrio* spp.) Strain/Administration Route/Dosage (CFU)	Phage Name or Acronym/Administration Route/Dosage (PFU)	Fish	Reference
*V. harveyi* VH2 from Hellenic Center for Marine Research Collection/immersion/10^6^–10^7^/mL of water	Virtus/immersion/10^8^/mL of water repeated twice	Gilthead seabream larvae	[46]
*V. harveyi* MM46 from Hellenic Center for Marine Research Collection/immersion/10^6^–10^7^/mL of water	vB_VhaS_MAG7/immersion/10^8^/mL of water	Gilthead seabream larvae	[43]
*V. anguillarum* PF4 isolated from diseased fish/immersion/5 × 10^5^/mL of water	CHOED/immersion/5 × 10^6^–10^8^/mL of water	Salmon	[47]
*V. anguillarum* isolated from aquaculture environment/immersion/10^6^/mL of water	VP-2/immersion/10^8^/mL of water	Zebrafish larvae	[9]
*-*	vB_Pd_PDCC-1/immersion/1.41 × 10^10^/mL of water	Longfin yellowtail larvae	[54]
*V. anguillarum* isolated from aquaculture environment/immersion/0.5–1 × 10^6^/mL of water	KVP40//immersion/0.5–12 × 10^8^/mL of water	Cod and turbot larvae	[55]

#### 3.1.3. *Edwardsiella*

*Edwardsiella* spp. is a Gram-negative, short rod-shaped intracellular bacterium that infects commercially important fish species, including turbot, channel catfish, tilapia, and flounder. It is a significant fish pathogen that enters through the gut and causes edwardsiellosis and enteric septicemia, leading to high mortality rates among economically important cultured fish species and considerable economic losses in the global aquaculture industry. Unfortunately, only a limited number of studies report in vivo experiments with bacteriophages against *Edwardsiella* spp.

*Edwardsiella piscicida*, previously known as *Edwardsiella tarda*, is the major pathogen among *Edwardsiella* spp. It induces severe edwardsiellosis, characterized by ascites and bacterial septicemia, accompanied by necrosis and ulceration in internal organs (kidneys, liver, spleen, and muscles). A lytic phage, ETP-1 (Podoviridae), first isolated from a marine fish farm, was characterized for its effectiveness against *E. piscicida*. It demonstrated MOI-dependent activity against MDR pathogenic *E. piscicida* [58]. The bacteriophage, stable across a wide range of temperatures and pH, proved effective in vivo in the zebrafish model. Administration by immersion (at approximately 10^8^ PFU/mL of water) resulted in a 68% increase in survival after a challenge by intraperitoneal injection with pathogenic *E. piscicida* (about 10^5^ CFU/fish) [58]. The same research group further explored the promising approach of bioencapsulating phage ETP-1 (by immersion at doses ranging from 10^5^ to 10^11^ PFU/mL) in the primitive crustacean brine shrimp (*Artemia*), which served as a feed delivery vector for the efficient administration of the phage to zebrafish. The bacteriophage disseminated in various organs of Zebrafish, even in the absence of any carrier, without adversely affecting immune stimulation [59]. Another bacteriophage specific to *E. piscicida* was isolated and tested in vivo to protect turbot, a species highly susceptible to *E. piscicida*. This lytic, narrow-tropic phage, vB_EtaM_ET-ABTNL-9, was tested in vivo through feeding, injection, or immersion (at MOIs of 1, 10, and 100) and significantly reduced mortality in turbot after intraperitoneal injection with the pathogenic bacterium (6 × 10^6^ CFU/fish). The best results were observed with immersion [60]. Although the phage triggered an immune response in turbot, it did not interfere with fish growth [60]. More recently, phage EPP-1 was isolated, characterized, and tested in vitro and in vivo against *E. piscicida* (in the zebrafish model) [61]. It exhibited inhibition of *E. piscicida* growth in vitro, comparable to the use of an antibiotic (florfenicol), and showed an increase (about 17%) in survival in vivo when administered by immersion at 1 MOI to zebrafish challenged intraperitoneally with 10^5^ CFU/fish [61]. Moreover, despite carrying genes for the lysogenic cycle, phage EPP-1 exhibited robust lytic activity, and treatment significantly reduced the presence of the floR resistance gene in zebrafish excreta and aquaculture water. This suggests that phage therapy may serve as a viable alternative to antibiotics, potentially reducing the spread of antibiotic resistance genes in aquaculture environments [61].

Another significant *Edwardsiella* spp. pathogen in aquaculture, particularly for catfish, is *E. ictaluri*. Two separate research groups isolated and studied bacteriophages targeting *E. ictaluri* in vitro; however, neither conducted in vivo experiments. A virulent phage named MK7 (Myoviridae) was isolated and characterized in vitro for features favorable to bacteriophage therapy. It demonstrated lytic activity, short latency, high specificity, and narrow host-range tropism (limited to a few strains of *E. ictaluri*) [62]. More recently, another research team isolated two lytic *E. piscicida* phages, vB_EiM_PVN06 (PVN06) and vB_EiA_PVN09 (PVN09), which belong to the families Myoviridae and Autographiviridae, respectively. Notably, PVN09 was also able to cross-infect *Escherichia coli* [63]. Based on their stability and life-cycle characteristics, both phages could be further studied for potential application in bacteriophage therapy against *E. ictaluri* [63].

The **phage cocktail** approach has only been developed for *E. piscicida*. A two-phage cocktail, combining vB_EpM_ZHS (Myoviridae) and vB_EpP_ZHX (Podoviridae), significantly suppressed bacterial proliferation in vitro, enhanced survival rates, and reduced both bacterial load and mortality rates by 30% and 55–75%, respectively, in zebrafish larvae and turbot in vivo models. In particular, zebrafish larvae were treated with 10^9^ CFU/mL of water and 10^9^ PFU/mL of water by immersion, while turbot were treated with 2 × 10^8^ CFU/mL of water and 2 × 10^7^–10^9^ PFU/mL of water by immersion. The phage stimulated an immune response in turbot, while zebrafish larvae were unable to eliminate the phages, likely because they lack an adaptive immune system. This suggests that adaptive immunity may play a critical role in clearing phages [64].

#### 3.1.4. *Streptococcus*

*Streptococcus* spp. is a Gram-positive bacterium that causes streptococcosis, a disease recognized as catastrophic for over 60 years in freshwater and saltwater aquaculture. In particular, infection in tilapia results in mass mortality rates of over 50%, with symptoms including septicemia, meningoencephalitis, irregular swimming, loss of buoyancy control, and spinal abnormalities. Streptococcosis is also common in finfish aquaculture, affecting species such as striped bass and dolphins. Vaccination is an effective and safe strategy against Streptococcus infections; however, this pathogen can persist within immune cells without eliciting systemic immune responses. Bacteriophages, as effective biocontrol agents, can be used for both preventive and therapeutic interventions against bacterial diseases. Among the microorganisms responsible for streptococcosis in aquaculture, *Streptococcus agalactiae* is the primary agent, followed by *Streptococcus iniae*. *Streptococcus agalactiae* infects a variety of aquatic organisms, including tilapia, golden pompano, ya-fish, gigantic Queensland grouper, frogs, crabs, and penaeid shrimp [65,66,67].

Although the isolation and characterization of bacteriophages against *S. agalactiae* date back to 1969 [68], practical applications were developed only recently, with in vivo experimental infections using the previously isolated HN48 phage in tilapia [65]. The HN48 bacteriophage, characterized by a broad yet highly specific host range, is stable at alkaline pH and temperatures up to 60 °C. It proved efficacious in vivo in tilapia when administered by intraperitoneal injections at an MOI of 1, resulting in about a 60% higher survival rate and delayed mortality after a challenge via intraperitoneal injection with pathogenic *S. agalactiae* (1.2 × 10^5^ CFU/fish) [65]. More recently, four lytic bacteriophages belonging to the Myoviridae and Siphoviridae families were identified and named 12P, 15F, 16E, and 20D [66]. These phages demonstrated a convenient, stable, and safe profile in vitro, characterized by a limited host range, significant reproductive capacity, and resilience to various physicochemical conditions, including temperature, pH, and salinity [66]. These phages demonstrated also a lack of lysogeny and transduction potential [66].

*Streptococcus iniae* causes streptococcosis primarily in finfish aquaculture. In the absence of vaccines and potentially effective bacteriophages, the growing emergence of antibiotic resistance may be addressed by phage-derived or synthetic **endolysins**. Endolysin therapy, a subset of bacteriophage therapy, involves the administration of cell wall-cleaving enzymes (endolysins) derived from bacteriophages. A study evaluated the effectiveness of three synthetic endolysins, PlyGBS 90–1, PlyGBS 90–8, and ClyX-2, in treating experimental *S. iniae* infections (3.5 × 10^7^ CFU/fish intraperitoneally injected) in hybrid striped bass [67]. All three endolysins demonstrated effective lysis of all six *S. iniae* strains, with ClyX-2 exhibiting an eightfold increase in bacteriolytic activity. Future research is needed to optimize endolysins-containing-feed formulations or investigate preventive water treatments as a cost-effective method of application [67].

#### 3.1.5. *Flavobacterium*

*Flavobacterium* spp. is a rod-shaped, Gram-negative, motile, or non-motile bacterium generally nonpathogenic; however, certain species can cause highly lethal diseases in freshwater fish, such as rainbow trout and other salmonids. In particular, *Flavobacterium columnare* can cause Columnaris Disease, *Flavobacterium psychrophilum* Bacterial Cold-Water Disease, and Rainbow Trout Fry Syndrome. Phage therapy has been suggested as an alternative approach for managing *Flavobacterium* infections in aquaculture. Table 5 summarizes, for the in vivo studies, the dosages of bacteria and phages administered via different routes in various fish.

*Flavobacterium columnare* is responsible for epidermal infections in farmed fish worldwide, leading to high mortality rates [69]. The disease is primarily epidermal and transmitted through water, affecting the skin, fins, and gills of fish [70]. In an early study, the previously isolated and characterized bacteriophage FCL-2 was tested in vivo by immersion and demonstrated efficacy in two different aquaculture species (rainbow trout and zebrafish) in preventing *F. columnare* infection [70]. Furthermore, the treatment significantly increased survival in both species, with 100% survival in zebrafish and 50% survival in rainbow trout. A single administration of the phage in small-scale experimental systems (inflow-through tanks) was sufficient to rescue the rainbow trout population [70]. Phage FCL-2, administered by immersion, also significantly increased survival in Atlantic salmon after experimentally challenging the yolk sac fry with *F. columnare* [71]. Additionally, the phage treatment reduced the pathogen in the water without affecting the α-diversity or the bacterial community composition of the rearing water [71].

*Flavobacterium psychrophilum* is responsible for high juvenile mortality and increased susceptibility to secondary infections [72]. In an early study that isolated and characterized bacteriophages targeting *F. psychrophilum* in vitro, phage PFpW-3 (Myoviridae) demonstrated high infectivity and lytic activity, along with adequate stability under variations in water pH and temperature. It also efficiently reduced bacterial multiplication in vitro [73]. However, the evolution of *F. psychrophilum*-resistant variants within just two days discouraged the use of this bacteriophage alone, suggesting that it would be more effective as part of a phage cocktail [73].

Two fundamental aspects of *Flavobacterum* spp. are the diversity of variant strains that can cause disease on one side and the bacteriophage-driven evolutionary pressure that leads to the rapid emergence of new variants on the other side. Therefore, gaining a deeper understanding of bacterial virulence traits and elucidating bacterial–phage interactions and dynamics in aquaculture are crucial. An extensive collection of complete genome sequences, annotations, and comparisons has been made available for the phages that infect *F. psychrophilum* [74]. The same research team further expanded the library of *F. psychrophilum*-specific bacteriophages and explored the mechanisms and implications of phage resistance. Their finding revealed that phage resistance in *F. psychrophilum* was associated with mutations causing reduced or lost motility, surface adhesion, and proteolytic activity. In some cases, resistance also led to a lack of virulence against rainbow trout fry [75]. Interestingly, reverse mutations restored phage susceptibility, motility, proteolytic activity, and virulence, suggesting that phage cocktails containing strains from different genomic clusters could reduce the risk of escape mutants [75].

The in vitro investigation of three phages, FpV4, FpV9, and FPSV-S20, demonstrated their efficacy as biocontrol agents against *F. psychrophilum* and facilitated the development of an efficient method for the rapid isolation of newly evolved bacteriophages with a broad spectrum of activity against *F. psychrophilum* [72]. The method, based on comparative genomic analysis of the mutations arising after serial phage–host range incubations, enables enhanced selection and isolation of new lytic phages in a short time. These phages can counteradapt to bacterial hosts that have evolved resistance or broaden their host spectrum in a manner far more specific than synthetic biology approaches [72].

A large collection of isolated and characterized bacteriophages has been made available also for *Flavobacterium columnare* [76]. It was demonstrated that bacteriophages isolated from geographically, temporally, and host-divergent sources exhibit a high degree of genetic homology [77]. Additionally, it was noted that more recently isolated phages have a broader host range compared to those isolated in the past, while bacteria isolated most recently were resistant to previously isolated phages [77]. This suggests that bacteria evolve bacteriophage resistance under aquaculture conditions and that the aquatic environment does not act as a barrier for bacteria and phages. The same research team also evaluated in vitro the development of resistance in *F. columnare* to the lytic Myophage FCV-1 in collected lake water. They found that phage resistance appeared within 24 h, followed by multiple subsequent co-evolutions [78]. Concomitant mutations in the phage occurred, allowing the virus to regain infectivity [78]. This method enables the rapid isolation of mutated, more effective phages that can overcome resistance, making them suitable for therapeutic applications.

Studies were conducted in both rainbow trout and rainbow trout fry to compare various **phage delivery methods** and evaluate their impact on controlling *F. columnare* and *F. psychrophilum* infections. In rainbow trout fry, a series of in vivo experiments were carried out to assay the optimal dose, compare single phages (FCOV-S1, FCOV-S2, and FCL-2) versus a three-phage-cocktail, and evaluate different phage administration routes, including bathing, phage-coated plastic sheets, and oral delivery via feed, both before and after infection with *F. columnare* [69]. The results indicated that the most effective approach to reducing mortality associated with columnaris disease in both stagnant and flow-through water systems was immersion [69]. This outcome may be attributed to the epidermal nature of the disease and the environmental route of transmission. However, phage-coated plastic sheets significantly delayed the onset of the disease in flow-through water systems. Oral administration initially accelerated disease progression, but by the end of the experiment, mortality was lower [69]. Regarding the use of a single phage versus a phage cocktail, the study found that efficacy depended on the specificity of the phage–bacterium interaction, regardless of the number of phages or the timing of treatment [69]. A similar in vivo experiment was conducted in juvenile rainbow trout by another research group to compare the administration of the single bacteriophage FpV-9 against *F. psychrophilum* via immersion with two different oral systems (stomach intubation and phage-coated feed) [79]. The results demonstrated the efficacy of all three systems (at optimized doses) in delivering the phage in a wide range of organs, with the phage-coated feed pellet being particularly effective in successfully delivering and maintaining bacteriophage density across various organs [79]. However, the same research team later showed that oral administration of a two-phage mix (FpV4 and FPSV-D22) via feed to rainbow trout fry altered the fish gut microbiota, regardless of the presence of *F. psychrophilum*. Despite these changes, no negative impact on fish growth or health was observed [80]. Since *F. psychrophilum* can form biofilms on aquaculture surface materials, the same researchers previously utilized the FPSV-D22 lytic bacteriophage to target *F. psychrophilum* biofilms. They demonstrated its ability to inhibit bacterial attachment and colonization, prevent biofilm formation, and disrupt mature biofilm [81]. Additionally, they determined the minimal phage-to-host ratio required for in vitro efficacy against biofilms. Following this, they conducted an in vivo experiment, which showed that a phage cocktail of FPSV-D22 and FpV4, when administered via intraperitoneal injection, significantly reduced (in a dose-dependent manner) mortality caused by *F. psychrophilum* in juvenile rainbow trout that had been experimentally infected via intraperitoneal injection [81]. Finally, the same research group compared different bacteriophage delivery methods, including oral (feed-sprayed and feed-immobilized phage), immersion, and injection, for administering a two-phage cocktail (FpV4 and FPSV-D22) to rainbow trout fry to protect against *F. psychrophilum*. The results showed that intraperitoneal injection was the most effective delivery route, emphasized the importance of dosage, and demonstrated that while oral administration allowed the phage to reach the gut, it was less effective in reaching other organs [82]. The effectiveness of intraperitoneal injection in delivering and maintaining the persistence of the bacteriophage (FpV-9) in various organs of rainbow trout was previously demonstrated by another research team. They also showed that phage persistence in the organs was higher when the bacteriophage was injected alongside the target *F. psychrophilum* bacteria [83]. The Recirculating Aquaculture System was also tested as a phage delivery method. RAS was applied on a rainbow trout farm, and the phage FCL-2, targeting *F. columnare*, was introduced into the water. The results showed that phages could persist in the RAS for at least three weeks after a single administration, without negatively affecting water quality or causing fish mortality [14]. The bacteriophages were preferentially adsorbed onto plastic carrier media, biofilm, and fish mucus, which may enhance phage persistence by enriching the phages and gradually releasing them [14].

**Table 5:** Summary of dosages of bacteria and phages administered via different routes to various fish against *Flavobacterium* spp.

Bacterium (*Flavobacterium* spp.) Strain/ Administration Route/Dosage (CFU)	Phage Name or Acronym/Administration Route/Dosage (PFU)	Fish	Reference
*F. columnare* B185 from diseased fish/immersion/3 × 10^6^/mL of water	FCL2/immersion/3 × 10^8^–10^7^/mL of water	Zebrafish and rainbow trout	[70]
*F. columnare* FC7/immersion/10^7^/mL of water	FCL2/10^7^/immersion/10^7^/mL of water	Atlantic salmon	[71]
*F. columnare* FCO-S1, FCO-S2, and B185 isolated from diseased fish/immersion/5 × 10^3^–1.5 × 10^6^/mL of water	FCOV-S1, FCOV-F27, and FCL-2/immersion-oral/5 × 10^2^–10^4^/mL of water-12.5–6 × 10^6^/g of feed	Rainbow trout fry	[69]
*F. psychrophilum* 950106-1/1 isolated from diseased fish/immersion/10^6^/mL of water	FPV-9/immersion-oral/10^8^/mL of water-10^8^/fish via intubation-2.5 × 10^7^/fish via feed	Rainbow trout	[79]
*F. psychrophilum* FPS-S6, 160401-1/5N, FPS-R9, and 950106-1/1 isolated from diseased fish/intraperitoneal injection/8 × 10^7^/fish	FPSV-D22 and FpV-4/intraperitoneal injection/1.2–2.2 × 10^4^–10^8^/fish	Rainbow trout	[81]
*F. psychrophilum* 950106-1/1 isolated from diseased fish/intraperitoneal injection/0.5–5 × 10^3^–1.7 × 10^6^/fish	FPSV-D22 and FpV-4/oral sprayed–oral immobilized–immersion–intraperitoneal injection/4.8–16 × 10^7^/g of feed—2.5–8.3 × 10^7^/g of feed—0.3–6 × 10^5^/mL of water twice—1.7 × 10^7^/fish	Rainbow trout fry	[82]
*F. psychrophilum* 950106-1/1 isolated from diseased fish/intraperitoneal injection/10^4^/fish	FpV-9/intraperitoneal injection/10^7^/fish	Rainbow trout	[83]
-	FCL-2/immersion/10^7^/mL of water	Rainbow trout	[14]

#### 3.1.6. *Citrobacter*

*Citrobacter* spp. is a Gram-negative, aerobic, or facultative anaerobic opportunistic bacterium widely distributed in nature. It is responsible for infections in aquaculture species, leading to co-morbidity between humans and fish. Finfish, crayfish, catfish, stingrays, angelfish, tilapia, carp, doctor fish, and eels can all be infected with *Citrobacter* and may show clinical manifestations such as lethargy, reduced mobility, atypical swimming behavior, and gill erythema or lesions in the tail or fin areas. However, to date, only two studies have aimed at identifying bacteriophages effective against *Citrobacter* spp. One study identified two phages, Citrophage MRM19 and Citrophage MRM57 (Sydoviridae and Podoviridae, respectively), and tested both in vitro and in vivo in a zebrafish model [84]. The phages, characterized by rapid absorption time, short latency, stability under temperature and pH variations, and lack of lysogeny, induced a significant decrease in bacterial counts in vitro and increased survival in challenged zebrafish (by immersion with 10^10^ CFU/mL) in vivo. The survival rate was 17%, 23%, and 26%, respectively, for the two phages individually or in a cocktail administered at 10^5^ PFU/mL [84]. Among the various species, *Citrobacter freundii* is the most pathogenic in aquaculture systems, associated with high mortality in tilapia, hemorrhagic septicemia, enteritis, kidney and gill lesions in catfish, systemic infections in carp, and gastroenteritis in rainbow trout. A new lytic bacteriophage, IME-JL8 (Siphoviridae), was isolated, characterized, and evaluated in vitro and in vivo [85]. This phage, also characterized by rapid absorption time, short latency, stability under temperature and pH variations, and lack of lysogeny, showed strong lytic activity both in vitro and in vivo. It was also effective in disrupting biofilms and led to a 100% increase in survival in carp that were intraperitoneally injected with the phage (0.2 × 10^7^ PFU/fish) and subsequently challenged with intraperitoneally injected pathogenic *C. freundii* (10^6^–10^10^/fish), proving its potential for practical application [85].

#### 3.1.7. *Pseudomonas*

*Pseudomonas* spp. is a Gram-negative, rod-shaped bacterium widely distributed in the environment, including in waters, and is an opportunistic pathogen for both humans and animals, including fish. In fish, *Pseudomonas plecoglossicida* and *Pseudomonas aeruginosa* are associated with deadly diseases.

*Pseudomonas plecoglossicida* causes hemorrhaging in ayu, particularly in Japan. The first successful attempt to develop bacteriophage therapy against *P. plecoglossicida* dates back over two decades when specific phages were isolated from diseased ayu, characterized in vitro, selected, and then tested in vivo by oral administration (10^7^ PFU/g of feed) in ayu experimentally infected via the same route with *P. plecoglossicida* (10^7^ PFU/g of feed) [86]. The presence of the phages PPpW-3 and PPpW-4 (Myoviridae and Podoviridae, respectively) in freshwater decreased bacterial growth and resulted in lower and delayed mortality when orally administered through feed in ayu [86]. A subsequent in vivo study reported by the same research group demonstrated that when the two bacteriophages (PPpW-3 and PPpW-4) were orally administered through impregnated feed (2.4 × 10^7^/fish) in a cocktail formulation, the protective activity increased substantially, and the mortality of ayu experimentally infected with pathogenic *P. plecoglossicida* (orally at 6.6 × 10^7^ CFU/fish, or by intramuscular injection at 0.6–1.6 × 10^4^/fish) decreased to about 20% (compared to 40–53% with single phages) [87].

*Pseudomonas aeruginosa* is known for its antibiotic resistance, particularly to β-lactamases. Some β-lactamase-resistant strains of *P. aeruginosa* have been isolated from catfish species, where they cause ulcerative lesions [88]. Lytic bacteriophages targeting *P. aeruginosa* have been isolated, characterized in vitro, and tested in vivo (topically administered at 10^10^ PFU/mL to the skin with a cotton swab), showing effectiveness in treating the ulcerative lesions in infected catfish [88].

More recently, *Pseudomonas fluorescens* has spread to a wide range of fish species, causing fin or tail rot with erosion of the infected areas and anal or internal hemorrhages. As previously reported for *Aeromonas* spp., the bacteriophage cocktail BAFADOR^®^, including seven bacteriophages, four of which target *Pseudomonas fluorescens*, demonstrated both prophylactic and therapeutic value when administered by immersion (10^5^/mL of water) to rainbow trout challenged by intraperitoneal injection with the pathogenic bacterium [37].

#### 3.1.8. *Yersinia*

*Yersinia* spp. is a Gram-negative, cocco-bacillary enterobacterium, including a species, *Yersinia ruckeri*, which is pathogenic for fish, especially salmonids, and in particular rainbow trout. It causes enteric red mouth disease and hemorrhagic septicemia, both of which are responsible for significant economic losses in aquaculture. A lytic phage (φNC10) targeting serotype O1 of *Yersinia ruckeri* was identified and characterized, and its virions exhibited polysaccharide depolymerase activity that degrades *Y. ruckeri* O1 lipopolysaccharides, rendering the pathogen serum-sensitive and neutralizing its virulence. This bacteriophage, when intraperitoneally injected (1–10,000 MOI), reduced mortality in rainbow trout challenged with *Y. ruckeri* (10^6^ CFU/fish by intraperitoneal injection), showing potential for application in salmonid aquaculture systems. φNC10 was found to attack only serotype O1 strains of this pathogen and therefore has a relatively narrow host range. However, since the majority of *Y. ruckeri* outbreaks are caused by this serotype, this will likely not limit its use as a therapeutic agent against this disease [89].

#### 3.1.9. *Plesiomonas*

*Plesiomonas* spp. is a Gram-negative, rod-shaped, facultative anaerobic enterobacterium that is ubiquitously present in nature, including the species *Plesiomonas shigelloides*, which can infect humans and other animals but is highly pathogenic for aquatic animals, particularly fish. The spread in aquaculture typically occurs in summer, during high-temperature waters, and symptoms include body surface swelling, eyeball protrusion, swollen and red anus, ascites in the abdominal cavity, microhemorrhages in the liver, and enlarged spleen. The lytic phage PSP01 (Siphoviridae) was isolated, characterized, and tested (intraperitoneally injected at 10^8^ PFU/fish twice) in grass carp challenged by intraperitoneal injection with *P. shigelloides* (10^8^ CFU/fish), showing over a 30% increase in survival and alleviated symptoms. The lytic mechanism was elucidated to be due to the presence of a lysis cassette made up of three genes, which are co-expressed for a synergic lytic effect [90].

#### 3.1.10. *Photobacterium*

*Photobacterium* is a marine, motile, psychrotrophic, facultative Gram-negative bacterium commonly found on the surfaces and in the intestinal contents of marine animals. *Photobacterium damselae* subsp. damselae is a pathogen capable of infecting multiple species of farmed fish, such as the Australian longfin eel, European seabass, gilthead seabream, meager, ovate pompano, rainbow trout, red-banded seabream, turbot, white seabream, and yellowtail. The lytic phage vB_Pd_PDCC-1 was isolated, characterized, and tested by immersion (2 × 10^8^ PFU/mL of water) in longfin yellowtail eggs challenged with *P. damselae* subsp. damselae (8.5 × 10^6^ CFU/mL of water). vB_Pd_PDCC-1 has a wide host range, including *P. damselae* subsp. damselae, *Vibrio alginolyticus*, *V. campbellii*, and *V. parahaemolyticus*, and its administration resulted in a significant reduction in *Vibrio* spp. load during hatching, reaching undetectable levels [91].

### 3.2. Phage Therapy in Mollusk Culture

Bacteriophage therapy may also be effective in treating bacterial infections in mollusk species, with the most targeted bacteria being *Vibrio* spp., followed by *Escherichia coli* and *Salmonella enterica*. The most commonly affected animals are oysters and abalones.

As mentioned earlier, the spread of *Vibrio* spp. in marine, estuarine, and freshwater environments is responsible for vibriosis, a devastating disease that can cause up to 100% mortality in contaminated facilities, not only in fish but also in shellfish. These shellfish can pass the pathogen to humans through the consumption of raw or improperly cooked seafood [11]. Among the numerous infectious *Vibrio* species, *Vibrio parahaemolyticus* is considered one of the primary foodborne pathogens worldwide, while *Vibrio coralliilyticus* and *Vibrio tubiashii* are responsible for high mortality rates in oysters, leading to significant economic losses globally [11]. The approach to managing these species varies depending on the goal: for *V. parahaemolyticus*, research primarily focuses on using phages to purify oysters and ensure their safety for human consumption. In contrast, for *V. coralliilyticus* and *V. tubiashii*, phage therapy is aimed at safeguarding the health of oysters and other mollusk species.

Apart from the public health impact, an outbreak of *V*. *parahaemolyticus* has significant social repercussions for the entire shellfish-producing region. Additionally, *V*. *parahaemolyticus* strains infecting seafood have shown resistance to multiple commercial antibiotics, similar to strains infecting humans. Therefore, the potential of various candidates for bacteriophage therapy has been investigated both in vitro and in vivo. The virulent bacteriophage pVp-1 (Siphoviridae), which infects a multi-antibiotic-resistant strain of *V*. *parahaemolyticus*, was tested in vivo by immersion or surface treatment on oysters previously challenged by immersion or surface spotting with the pathogen in an artificial system. The results showed that bacterial growth was significantly diminished in the case of immersion and completely inhibited for surface-applied phage [92]. The lytic phage vB_VpaS_OMN (Podoviridae) demonstrated in vitro efficacy by markedly reducing (up to 99%) the bacterial count when administered to the surface of oysters or to the oyster meat artificially contaminated with the bacterium [93].

Although oyster hatcheries comply with strict hygienic measures, multiple preventive strategies are desirable to limit pathogenic bacteria contamination and improve overall oyster hatchery production. One such strategy could involve bacteriophage treatment of microalgae, which serve as the primary food source for oyster larvae during hatching and, as a result, may act as a vector for *Vibrio* spp. infection. In vitro, a cocktail of bacteriophages from the Myoviridae family, applied to antibiotic-resistant vibrio-contaminated microalgae suspensions, significantly reduced the bacterial count. This suggests that bacteriophages may be a viable approach for eliminating *Vibrio* spp. contamination in oyster and other bivalve mollusk hatcheries through feed vectors [94]. Another promising strategy involves the use of bacteriophages in the depuration process of oysters to effectively reduce pathogens. For this purpose, the single lytic bacteriophage VPp1, targeting V. *parahaemolyticus*, was tested in vivo in live oysters experimentally contaminated with the pathogen. The results demonstrated a substantial decrease in pathogen levels, confirming the depurative potential of the phage [95].

*V. parahaemolyticus* is also highly efficient at forming mature biofilms on surfaces used in seafood processing, which increases the bacteria’s resilience and the associated risks. The phage CAU_VPP01 (Siphoviridae) demonstrated excellent lysis of planktonic cells and eradication of biofilms of *V. parahaemolyticus* in vitro, both on inanimate surfaces and on seafood surfaces, such as those of mollusks (squid) and fish [96]. This suggests that bacteriophages could be employed as a control method in combination with other sterilization techniques.

*Vibrio coralliilyticus* and *Vibrio tubiashii* are responsible for high larval oyster mortalities in mollusk hatcheries, particularly along the East and West American coasts. Sixteen new phages targeting *V. coralliilyticus* and *V. tubiashii* were recently isolated, characterized, and tested in combined phage cocktails on larval oysters to assess their potential in reducing mortality caused by these pathogens [97]. A five-phage cocktail (VTP100) significantly reduced mortality (by up to 91%) in 6 days in Eastern and Pacific oyster larvae, while a later-generation three-phage cocktail (VCP300) also reduced mortality by the same rate and was specific to eight different strains of *V. coralliilyticus* and *V. tubiashii* [97]. These results are particularly noteworthy because both strains are highly pathogenic, and the beneficial effect was much longer lasting than in other studies. Moreover, this approach could be applied on a large scale in hatcheries, especially for prophylactic purposes to prevent infections from establishing and proliferating within the larvae.

Other studies have been conducted on bacteriophages targeting *V. natriegens*, a pathogen capable of causing mortality in juvenile abalones, as well as in other mollusks and crustaceans. The phage vB_VnaS-L3 (Siphoviridae), isolated from *Vibrio natriegens*, was genetically analyzed and tested in vivo in abalone to assess its viability as a biocontrol agent against this pathogenic bacterium [98]. The results showed a survival rate of 95%, which was higher than that achieved with antibiotic treatment, suggesting that this phage could be considered a promising candidate for preventing *V. natriegens* disease outbreaks in marine aquaculture [98].

Abalone is also endangered by the withering syndrome, an infectious disease caused by an intracytoplasmic rickettsia-like organism known as Withering Syndrome Rickettsia-Like Organism (WS-RLO) or “Candidatus Xenohaliotis californiensis”, for which limited information is available. The disease affects black, green, pink, and red abalones, inducing morphological changes in the host’s digestive gland, impairing its function, and leading to the catabolism of foot muscle for energy, ultimately resulting in death. Phage RLO, identified in black abalone, reduced bacterial load and associated mortality [99]. The bacteriophage’s mechanism of action was atypical, not relying on the lysis of the target bacterium, but instead altering the course of the infection by proficiently disrupting the pathogen’s normal function. The presence of the phage increased the mollusk’s tolerance to the infecting bacterium, and the longevity of the abalone varied depending on exposure histories and infection tolerance [99].

Mollusks, particularly oysters and bivalves, may also be contaminated by enteric pathogens such as *Escherichia coli* and *Salmonella enterica*. A six-bacteriophage cocktail (including five *E. coli* phages and one *S. enterica* phage) was found to be effective against resistant strains of *E. coli* and *S. enterica* in edible oysters, drastically reducing the number of colonizing bacteria [100]. The use of a bacteriophage cocktail broadens the activity spectrum and overcomes bacterial resistance. Similarly, another research group isolated two new phages (phT4A and ECA2) targeting *E. coli* and tested them both individually and in a cocktail in vivo in edible cockles naturally contaminated (in static seawater) and artificially contaminated (in depuration system) with the aim of decontamination and purification [101]. The results showed a similar decrease in bacterial count in both naturally and artificially contaminated cockles when treated with either a single phage or the phage cocktail, similar to the decrease obtained with the regular industrial decontamination process. However, when the bacteriophage treatment was combined with the depuration procedures, a faster decrease in bacterial count was achieved, resulting in enhanced quality and safety of the bivalves intended for human consumption [101].

### 3.3. Phage Therapy in Crustacean Culture

Among the various crustacean species, shrimp is the most important for farming, with production intensifying due to significant worldwide demand and export opportunities.

Among the bacteria, *Vibrio* spp. is the leading cause of economic losses in the shrimp industry, followed by *Aeromonas* spp.

All stages of the shrimp life cycle, from eggs to broodstock, can be affected by *Vibrio* spp. infection, which typically results in 100% mortality in contaminated facilities [11,102]. The genus *Vibrio* includes a range of pathogenic species that impact not only the farming of fish and mollusks but also the farming of crustaceans. Therefore, alternatives to disinfectants and the limited number of allowed antibiotics are being explored, with bacteriophage therapy emerging as a promising option. Some phages target multiple *Vibrio* spp., while others are specific to certain highly pathogenic strains. Among the *Vibrio* species, the most pathogenic in crustaceans are *Vibrio parahaemolyticus*, *Vibrio harvey*, and *Vibrio alginolyticus*.

*Vibrio parahaemolyticus* is regularly monitored in shrimp hatcheries, not only because it is the primary cause of seafood-related bacterial gastroenteritis globally, but also because it causes necrosis, stunted growth, muscle opacity, anorexia, and, ultimately, death in crustaceans. Some strains are responsible for acute hepatopancreatic necrosis disease, also known as early mortality syndrome, which is characterized by mortality rates of up to 100% in marine shrimp aquaculture. An early study investigated the efficacy of two selected lytic phages, Vpms1 and A3S, in preventing and controlling *V. parahaemolyticus* in shrimp larvae [102]. The results demonstrated the efficacy of both phages (when administered individually) in reducing mortality due to *V. parahaemolyticus*, with efficacy proportional to the earliest administration time in gnotobiotic shrimp [102]. Another study reports the isolation and in vitro characterization of phage VVP1 (Myoviridae), which is specific against two strains of *V. parahaemolyticus* and can also infect two strains of *V. alginolyticus*. The phage, which is stable across a wide range of temperatures and pH values, was tested in vivo in a laboratory trial with challenged postlarval shrimp, showing enhanced survival rates [103]. A research group first isolated and characterized the phage pVp-1 (Siphoviridae) in vitro, which is highly pathogenic to various strains of *V. parahaemolyticus* [104]. The phage was subsequently tested in vivo for its therapeutic value in marine shrimp, demonstrating a significant reduction in mortality with bacteriophage treatment (50% mortality with feed administration and 20–50% by immersion) [105]. However, the preventive application was found more efficacious than the therapeutic use, confirming that the timing of treatment is crucial for the outcome of the disease [105]. Similar promising results were obtained with another lytic bacteriophage (vB_VpaS_PG07), also belonging to the family Siphoviridae. This phage exhibited in vitro bacteriolytic activity, a short latency period, and stability under varying temperatures and pH levels. In vivo, it was able to infect *V. parahaemolyticus* in shrimp, reducing mortality in a dose-dependent manner [106]. More recently, a new bacteriophage, vB_VpaP_SJSY21, was isolated and characterized in vitro. This bacteriophage efficiently infects *V. parahaemolyticus*, and showed thermoresistance, a short latent period, and the absence of toxic genes [107]. Its effect on shrimp gut microbiota was studied, revealing that the presence of this phage correlated with a change in the microbial community composition of the shrimp gut, resulting in a lower abundance of *V. parahaemolyticus* and a higher abundance of *V. harveyi* [107]. Since the proportion of a particular microorganism impacts the others and the entire microbial community, and given that the gut microbiota of shrimp influences their health, phage therapy may serve as an effective measure to prevent acute hepatopancreatic necrosis disease in the shrimp industry. Furthermore, since acute hepatopancreatic necrosis disease may be caused by various pathogenic strains of *V. parahaemolyticus*, developing bacteriophage cocktails effective against a broad range of *V. parahaemolyticus* strains is particularly beneficial. A research team developed a high throughput bacteriophage screening system to rapidly select phages virulent against specific bacterial strains. This method was applied to identify two vibriophages (Eric and Ariel) targeting virulent *V. parahaemolyticus* strains [108]. The cocktail of these two phages successfully suppressed bacterial growth both in vitro and in vivo (in shrimp by immersion), rescuing the shrimp from infection within one day. Additionally, the vibriophages were able to stably colonize the shrimp gut microbiota, thereby playing a prophylactic role in preventing further infection [108].

Bacteriophages specific to *V. parahaemolyticus* have also been isolated from inland saline shrimp farms. One phage (V5, Inoviridae), out of twelve initially isolated, was selected in vitro for its specificity against different strains of *V. parahaemolyticus* and not other bacterial species [109]. Its effectiveness in reducing *V. parahaemolyticus* infection was then evaluated in shrimp, showing a reduction of over 78% in bacterial count within one hour [109]. Unlike other phages considered for bacteriophage therapy, which typically have a dsDNA genome, this filamentous page has an ssDNA genome and does not lyse the host after virion release. Instead, viral particles are continuously shed, resulting in a stable chronic infection [109]. Further research could clarify the biotechnological significance of this unique feature.

Another species of Vibrio spp., *Vibrio campbellii*, is emerging as an opportunistic pathogen associated with luminous vibriosis and potentially linked to acute hepatopancreatic necrosis disease. It is often mistaken for *Vibrio harvey* due to molecular and phenotypic similarities. To selectively target this specific *Vibiro* species without harming beneficial microorganisms, two studies from the same research group identified and characterized lytic phages, vB_Vc_SrVc9 and vB_Vc_SrVc2, both specific to *V. campbellii* [110,111]. These phages demonstrated in vitro stability in high salinity environments and at temperatures ranging from 20 to 40 °C. In vivo, phage vB_Vc_SrVc9, when tested on shrimp challenged with pathogenic *Vibrio campbellii* increased survival by over 60% without negatively impacting the natural microbiota [110]. More recently, another research group isolated and characterized the lytic bacteriophage OPA17 (Siphoviridae), which targets several *V. campbellii* strains, along with *V. parahaemolyticus* and *V. vulnificus* [112]. Phage OPA17 showed a significant (70%) increase in shrimp survival when infected with *V. campbellii* and also reduced *V. campbellii* biofilm formation in vitro [112]. Although OPA17 could be an ideal candidate for phage therapy due to its broad lytic activity and biofilm reduction, the lysis of *Gram-negative* bacteria, which releases endotoxins, remains a concern.

*Vibrio harvey* is a prevalent cause of mortality in hatcheries and culture systems for larval shrimps, in addition to being a pathogen for fish, as mentioned above. A decade ago, the bacteriophage VHP6b (Siphoviridae) was isolated from mollusks, proven safe (lack of integrases), and tested for its ability to protect against *V. harveyi* in a simulated shrimp hatchery system [113]. It was found to be broadly pathogenic for *V. harveyi* and, when administered to the medium just before the challenge, improved shrimp postlarvae survival by 40 to 60% [113]. To reduce the risk of developing phage-resistant bacterial strains, a few years later, three additional phages targeting *V. harveyi* were isolated, characterized, and tested in a cocktail formulation in shrimp larvae microcosm [114]. The phages, VHM1-N2A and VHM2-N8A (Myoviridae), and VHS1-N10A (Siphoviridae), showed stability at temperatures between 4 and 50 °C and within a pH range of 4 to 10. The phages had a safe profile and a broad host range, targeting *V. harveyi*, *V. alginolyticus*, and *V. parahaemolyticus* [114]. When the cocktail was used, the survival of shrimp larvae in laboratory conditions increased to over 80%, compared to 60 to 78% with the individual phages [114]. More recently, the phage ΦLV6 (Siphovirus), which is lytic against various luminescent strains of *V. harveyi*, was isolated, characterized in vitro, and tested in vivo in glass tanks with shrimp postlarvae [115]. This bacteriophage significantly reduced both shrimp postlarvae mortality and bacterial count [113]. In another study, the addition of recombinant lysozyme to the phage formulation was shown to significantly improve the phage’s efficacy both in vitro and in vivo in microcosms. The enzyme facilitated the phage’s entry into the target bacterium, enhancing the overall treatment [116]. The wide host range, high growth rate, short latency period, and safety profile of these phages make them promising candidates for bacteriophage therapy.

As reported earlier for fish therapy, a jumbo lytic bacteriophage, vB_VhaM_pir03 (Myoviridae), was identified and described as a potential candidate for phage therapy against *Vibrio harveyi* due to its rapid adsorption, short latent phase, broad activity against *V. harveyi* and other antibiotic-resistant *Vibrio* species, and the absence of transduction potential, virulence, or antibiotic-resistance genes [45]. In vivo trials conducted on shrimp challenged with *V. harveyi* showed a significant reduction in mortality within 1 day [45].

*Vibrio alginolyticus* is another significant pathogenic species of Vibrio spp. that infects a variety of aquatic organisms, including crustaceans, mollusks, fish, and humans, where it causes skin and ear infections, as well as acute gastroenteritis. The bacteriophage vB_ValM_PVA23 (PVA23, Myoviridae) was isolated and characterized in vitro, showing lytic activity against pathogenic strains of *V. alginolyticus* and demonstrating safety and stability across temperature and pH variations [117]. Further in vivo trials conducted in laboratory settings and shrimp farms revealed a rapid and significant reduction in *V. alginolyticus* counts, outperforming chemical iodine disinfectants, with negligible rebound effects [117]. At the same time, the same research group isolated another bacteriophage, vB_ValM_PVA8 (PVA8, Straboviridae), which effectively infects both *V. alginolyticus* and *V. parahaemolyticus* pathogenic strains [118]. This broad-range host phage, stable under temperature and pH variations and lacking lysogenic or resistance and integrating genes, was tested in vivo on shrimp infected with *V. parahaemolyticus*, resulting in a survival rate of about 89% [118]. Additionally, in shrimp farm trials, the bacteriophage reduced the counts of both *V. alginolyticus* and *V. parahaemolyticus* in the water, demonstrating its biological effectiveness in controlling *Vibrio* infections in commercial shrimp farming plants [118].

A specific approach was developed using two lytic bacteriophages, φSt2 and φGrn1, which are specific for *V. alginolyticus*. These phages were administered in a cocktail formulation to live prey such as the crustacean *Artemia salina* to protect aquaculture species from *V. alginolyticus* infections. The results from both in vitro and in vivo tests (showing a 93% reduction in *Vibrio* counts) suggest that live-feed animals like *Artemia* are effective phage vectors for regulating Vibrio levels in marine hatcheries [52].

As previously described for other bacteria, the use of a single phage typically leads to the rapid development of resistance to the therapy, while phage cocktails help delay the evolution of resistance. The co-evolutionary dynamics of bacteria–phage interactions have been experimentally evaluated with *Vibrio* spp. and three lytic phages, V1G, V1P1, and V1P2 [119]. The study showed that administering single phages resulted in the rapid development of bacterial resistance, similarly for all three phages, with only slight temporal differences. This also led to a decrease in phage infectivity, such that no stable co-evolutionary pattern could be observed, and the phages did not regain infectivity [119]. Consequently, bacteria became more resistant to future phages. In contrast, using a cocktail of bacteriophages resulted in much lower bacterial resistance, which took significantly longer to develop. The infectivity of the phages remained high throughout the experiment [119]. These findings were supported by two more recent studies. One study focused on optimizing an efficacious bacteriophage treatment against *Vibrio* spp. It showed that a cocktail of five broad-host-range lytic phages (Siphoviridae) inhibited the growth of pathogenic bacteria in vitro more efficiently and for a longer period compared to single bacteriophages, while also increasing the survival rate of shrimp in vivo [120]. Another study used the two phages previously employed individually (vB_Vc_SrVc2, and vB_Vc_SrVc9) to prepare a cocktail, which proved more efficient in selectively inhibiting several pathogenic *Vibrio* species in shrimp postlarvae. The cocktail also preserved other bacterial populations and caused less disruption to the intestinal microbiota [121]. These data highlight the importance of understanding co-evolutionary dynamics when considering bacteriophages for anti-bacterial therapy and emphasize the need to carefully select phages based on these dynamics.

*Aeromonas hydrophila,* in addition to being a significant problem for the fish industry, is also pathogenic for crustaceans, causing substantial economic losses, particularly in shrimp, due to mass mortality. Recently, the new Aeromonas-infecting bacteriophage AHPMCC7 was identified and characterized, demonstrating lytic activity both in vitro and in vivo on a laboratory scale [122]. The phage showed resilience across a wide range of pH, temperature, and salinity conditions in vitro, and in a glass aquarium system, it resulted in a significant reduction of *A. hydrophila* after 2 weeks of treatment, along with increased survival rates of juvenile shrimp [122]. The absence of virulence, integration, and antibiotic-resistant genes in the genome, along with the results from the small-scale trials, suggests that AHPMCC7 is a promising candidate for managing *A. hydrophila* in shrimp aquaculture.

### 3.4. Phage Therapy in Echinoderm Culture

The sea cucumber industry is rapidly growing, especially in Asia. As a result, some research has focused on phage therapy to combat antibiotic resistance in this type of farming, where *Vibrio* spp. is the most important pathogen. In particular, *Vibrio alginolyticus*, *V. cyclitrophicus*, and *V. splendidus* can cause skin ulceration syndrome, along with viscera ejection in juvenile sea cucumbers.

A preliminary study focused on *Vibrio alginolyticus* demonstrated that two newly isolated bacteriophages, PVA1 and PVA2 (Podoviridae and Myiviridae, respectively), which were virulent in single in vitro tests against pathogenic *V. alginolyticus*, increased the survival of challenged sea cucumbers by up to 73% (in an MOI-dependent manner) when administered in vivo in the marine environment in a cocktail formulation [123]. The same research group concurrently studied *Vibrio cyclitrophicus*, isolated the lytic bacteriophage vB_VcyS_Vc1 (Siphoviridae), and tested its efficacy in protecting sea cucumbers in the marine environment from *V. cyclitrophicus* [124]. The results, comparable to those obtained with the antibiotic treatment, showed increased survival of challenged sea cucumbers, ranging from 18 to 81% when the phage was incorporated into feed, 58% when injected, and 63% when delivered by immersion [124]. Finally, the same research team focused on *Vibrio splendidus*. Three bacteriophages (PVS-1, PVS-2, and PVS-3) were isolated and tested in vitro for virulence against *V. splendidus*. These phages were administered individually and as a cocktail incorporated into the feed of sea cucumbers for 60 days prior to the challenge [125]. The highest survival rate (82%) was recorded in echinoderms treated with the bacteriophage cocktail, which was equivalent to the survival rate achieved with antibiotic treatment. Lower survival rates were observed for single-phage treatments, ranging from 50 to 65%. The phage cocktail proved more effective than individual phages [125].

Although further research is needed for practical application, these results are promising for the use of bacteriophage therapy in the sea cucumber aquaculture industry as well.

## 4. Conclusions and Perspectives

Phage therapy is increasingly considered the most promising alternative to antibiotics in a “One-Health” approach to treating bacterial diseases. The aquaculture industry faces multiple challenges, including antibiotic resistance, the lack of necessary vaccines, and the formation of biofilms. Despite several decades of effort in vaccine development, effective universal commercial vaccines are still unavailable. Therefore, developing efficacious strategies to manage infectious diseases is crucial for the economic viability and sustainability of aquaculture. Aquaculture’s bacteriophage therapy has only gained popularity in recent years, and as a result, the number of studies available is fewer compared to other livestock sectors. The conditions to consider when applying phage therapy in aquaculture are varied and depend on the type of farming. For example, crustaceans have very different requirements compared to fish. Therefore, the stability of phages under certain conditions, such as water salinity, temperature, pH, and ultraviolet radiation, is much more stringent. As a result, many more studies focus on bacterial species that cause disease in fish than in mollusks and crustaceans, and even fewer on sea cucumbers.

The vast majority of the bacteriophages studied have been tested in vivo; however, for those tested only in vitro, it remains impossible to directly translate the results obtained in vitro to an in vivo system. In some cases, in vivo studies have been conducted exclusively on the zebrafish animal model. Another issue is that most of the phages used in aquaculture have not been fully characterized in terms of their genomic and proteomic features. As a result, there is limited information on their full potential and possible toxicity to the organism. Indeed, the major limitation to the application of bacteriophage therapy in aquaculture is the lack of comprehensive data, which in turn restricts the genetic engineering of the phages themselves. While both lytic and lysogenic phages, including genetically modified bacteriophages, have been considered for therapy in humans and other livestock animals, lysogenic and engineered phages are still largely neglected in aquaculture. Lysogenic phages, engineered to delete potentially pathogenic or integrating genes, may be more advantageous than lytic phages, especially when targeting Gram-negative hosts, which could release endotoxins upon cell lysis.

Another challenge is the rise of phage-resistant, mutated pathogenic bacterial strains. In this context, cocktails of multiple bacteriophages are the preferred option for combatting multidrug-resistant pathogens. As demonstrated by several comparative studies between single phages and phage cocktails, the latter has proven to be more efficacious in the vast majority of cases. Additionally, the delivery method also impacts the efficacy of phage therapy. Administration varies depending on the characteristics of the phage, the nature of the infection, and the animal species. In aquaculture, injection is generally the most effective method for targeting bacteria that cause clinical signs in internal organs, but this method is associated with a high mortality rate. Moreover, when animals are small and numerous, injecting phages can be labor-intensive and time-consuming. Therefore, phage-incorporated diets (phage-coated feeds) have been successfully used to treat large quantities of fish, ensuring continuous delivery of phages at designated times. Bacteriophages can be incorporated into any aquafeed source [126]. However, this method also has its drawbacks, such as the challenge of ensuring phage survival in the highly acidic and proteolytically active environment of the stomach. On the other hand, the more practical immersion method is not as effective as the other two, except for treating epidermal diseases.

In conclusion, phage therapy as an alternative to antibiotics for treating bacterial diseases in aquaculture is still in the early stages of development. It requires further understanding of the phage susceptibility of virulent bacterial strains, their genetic traits involved in pathogenicity, and the phage–bacterium co-evolution before it can be fully validated.

## Figures and Tables

**Table 1:** Summary of the most frequent bacterial infections and diseases in fish species [6].

Bacterium	Disease	Common Target
*Acinetobacter baumannii*	Ulcerations	Various fish
*Acinetobacter pittii*	Ulcerations	Various fish
*Aeromonas hydrophila*	Hemorrhagic Septicemia	Various fish
*Aeromonas salmonicida*	Furunculosis	Salmonids
*Aeromonas veronii*	Hemorrhagic Septicemia	Various fish
*Chryseobacterium balustinum*	Columnaris Disease	Various fish
*Citrobacter freundii*	Inflammation, Necrosis	Various fish
*Cytophaga columnaris*	Columnaris Disease	Various fish
*Edwardsiella ictaluri*	Enteric Septicemia	Catfish
*Edwardsiella piscicida*	Edwardsiellosis	Various fish
*Edwardsiella tarda*	Edwardsiellosis	Various fish
*Enterococcus faecalis*	Ulcers, Enteric Inflammation	Flatfish
*Flavobacterium columnare*	Columnaris disease	Various fish
*Flavobacterium psychrophilum*	Bacterial Cold Water Disease	Salmonids
*Francisella noatunensis*	Francisellosis	Atlantic cod
*Lactococcus garvieae*	Lactococcosis	Various fish
*Listeria monocytogenes*	Listeriosis	Various fish
*Moritella viscosa*	Winter Ulcer Disease	Salmonids
*Mycobacterium fortuitum*	Mycobacteriosis	Various fish
*Mycobacterium marinum*	Mycobacteriosis	Various fish
*Mycobacterium salmoniphilum*	Mycobacteriosis	Salmonids
*Nocardia asteroids*	Nocardiosis	Various fish
*Nocardia seriolqe*	Nocardiosis	Yellowtail, Seabass
*Photobacterium damselae*	Photobacteriosis	Various fish
*Piscirickettsia salmonis*	Piscirickettsiosis	Salmonids
*Plesiomonas shigelloides*	Hemorrhagic Ulcers	Various fish
*Pseudomonas aeruginosa*	Ulcers, Hemorrhagic Septicemia	Various fish
*Pseudomonas anguilliseptica*	Red Spot Disease	Eels, various fish
*Pseudomonas fluorescens*	Hemorrhagic Septicemia	Various fish
*Renibacterium salmoninarum*	Bacterial Kidney Disease	Salmonids
*Streptococcus agalatiae*	Streptococcosis	Tilapia, various fish
*Streptococcus iniae*	Streptococcosis	Various fish
*Streptococcus parauberis*	Streptococcosis	Various fish
*Streptococcus phocae*	Streptococcosis	Various fish
*Tenacibaculum maritimum*	Tenacibaculosis	Various fish
*Vibrio anguillarum*	Vibriosis	Various fish
*Vibrio harveyi*	Vibriosis	Various fish
*Vibrio kanaloae*	Vibriosis	Various fish
*Vibrio parahaemolyticus*	Vibriosis	Various fish
*Vibrio vulnificus*	Vibriosis	Various fish
*Vibrio splendidus*	Vibriosis	Various fish
*Yersinia ruckeri*	Enteric Redmouth Disease	Salmonids

**Table 2:** Summary of the most common bacterial co-infections in fish species [7].

First Pathogenic Bacterium	Second Pathogenic Bacterium	Common Target
*Aliivibrio wodanis*	*Moritella viscosa*	Atlantic salmon, *Salmo salar*
*Edwardsiella ictaluri*	*Aeromonas hydrophila*	Vietnamese catfish, *Pangasianodon hypophthalmus*
*Edwardsiella ictaluri*	*Flavobacterium columnare*	Thailand striped catfish, *Pangasianodon hypophthalmus*
*Renibacterium salmonarum*	*Aeromonas hydrophila*	Chinook salmon, *Oncorhynchus tshawytscha*

## Data Availability

No new data were created or analyzed in this study.
